# Cancer-Associated Parasitic Neglected Tropical Diseases: Precision Diagnostics, Risk Stratification, and Implementation Priorities

**DOI:** 10.3390/tropicalmed11100267

**Published:** 2026-09-26

**Authors:** Monica C. Botelho

**Affiliations:** 1 Department of Health Promotion and Non-Transmissible Diseases, INSA-National Institute of Health Dr. Ricardo Jorge, 4000-055 Porto, Portugal; monicabotelho@hotmail.com; 2 i3S-Instituto de Investigação e Inovação em Saúde, Universidade do Porto, 4200-135 Porto, Portugal

**Keywords:** parasitic neglected tropical diseases, schistosomiasis, molecular carcinogenesis, metabolic reprogramming, Warburg effect, precision diagnostics, digital pathology, geospatial surveillance, parasite-associated cancer, global health

## Abstract

Parasitic neglected tropical diseases (PNTDs) are primarily infections of poverty, impaired sanitation, and environmental exposure, but only a limited subset has recognized links to malignancy. The rationale for this Perspective is that these cancer-associated infections sit at the interface of communicable disease control, chronic inflammation, delayed organ damage, and non-communicable cancer prevention. At least 253.7 million people required preventive treatment for schistosomiasis in 2024, while infection-attributable cancer estimates suggest that liver flukes accounted for approximately 1300 to nearly 7000 new cholangiocarcinoma cases annually depending on modelling assumptions. The strongest recognized cancer associations involve *Schistosoma haematobium* with urinary bladder cancer and the food-borne liver flukes *Opisthorchis viverrini* and *Clonorchis sinensis* with cholangiocarcinoma. Importantly, infection with these parasites does not inevitably lead to cancer; malignant transformation is an uncommon, long-latency outcome shaped by parasite burden, reinfection, chronic inflammation, host susceptibility, co-infections, environmental exposures, nutrition, and access to care. This Perspective proposes a focused, risk-stratified framework that links parasite control, precision diagnostics, digital surveillance, targeted longitudinal follow-up, and implementation priorities for selected high-risk infections and settings. Rather than advocating broad cancer surveillance for all PNTDs, the article argues for staged, locally feasible approaches that strengthen existing control platforms while using advanced tools selectively for research, referral pathways, and populations at elevated risk.

## 1. Introduction

As the global health community works toward renewed neglected tropical disease control and elimination targets, the scientific and clinical framing of selected parasitic infections is undergoing an important transition. Most parasitic neglected tropical diseases should continue to be addressed primarily through prevention, diagnosis, treatment, sanitation, and transmission control. However, a small subset of parasitic infections has recognized links to long-latency malignancy. IARC classifies *S. haematobium*, *O. viverrini*, and *C. sinensis* as carcinogenic to humans, and epidemiological evidence links *S. haematobium* to bladder squamous cell carcinoma and liver flukes to cholangiocarcinoma [1,2]. At the global level, schistosomiasis remains a major public health problem, with at least 253.7 million people requiring preventive treatment in 2024, most of them in Africa [3]. For liver flukes, estimates of cholangiocarcinoma attributable to *O. viverrini* and *C. sinensis* vary substantially, from about 1300 new cases in one global infection-attributable cancer analysis to nearly 7000 annual cases in alternative modelling approaches [2,4]. These figures justify focused attention while also underscoring that cancer represents a minority, long-latency outcome rather than the expected consequence of infection.

The novelty of this Perspective is not to propose a new universal cancer-screening program for all PNTDs, but to integrate parasite control, precision diagnostics, implementation science, multimorbidity, and cancer-prevention thinking into a proportionate framework for the few parasitic infections with established carcinogenic relevance. This framework is intentionally limited to infections with recognized links to cancer risk, particularly *Schistosoma haematobium*, *Opisthorchis viverrini*, and *Clonorchis sinensis*. These pathogens are prioritized because they provide well-recognized examples of parasite-associated chronic pathology and malignancy, while also serving as model systems for understanding how persistent infection can reshape tissue microenvironments. Nevertheless, most infected individuals do not develop cancer, and infection should not be treated as a deterministic cancer precursor. Cancer risk depends on cumulative exposure, intensity and duration of infection, reinfection, chronic inflammation, host genetics, environmental cofactors, co-infections, nutrition, and access to timely diagnosis and treatment [1,2,3,4,5]. In this context, PNTD research and control should complement parasite clearance with risk-stratified assessment of tissue injury, fibrosis, and malignancy risk only in selected high-risk infections, populations, and health-system settings (Figure 1).

## 2. Precision Diagnostics and Biomarkers

The reliance on microscopy for detecting eggs in urine or stool is increasingly complemented by more sensitive diagnostic approaches. Circulating antigen assays, including tests for circulating anodic antigens, can identify low-intensity infections that may be missed by conventional parasitological methods [6,7,8]. These tools are especially important in areas approaching elimination, where residual transmission can persist at levels below the threshold of routine detection.

Metabolomics adds another layer to this diagnostic transition. Host-fluid metabolic signatures may reveal early tissue changes before overt clinical disease appears. In schistosomiasis-associated bladder and urogenital pathology, parasite-associated molecular and clinical changes have been discussed as potential indicators of chronic injury and malignancy-related risk [5,9,10]. Such biomarkers could support earlier risk stratification, particularly when combined with clinical staging and imaging.

Environmental DNA surveillance further expands the diagnostic field from the individual to the ecosystem. By detecting parasite DNA in water sources, public health teams can identify transmission hotspots without relying exclusively on repeated community-wide testing [11]. This approach is well aligned with precision public health, where interventions are targeted to the locations and populations at greatest risk.

Within this framework, it is important to distinguish diagnostics for active infection from biomarkers of tissue injury and biomarkers of cancer risk. Parasite detection tools identify current or recent infection, whereas host-response markers, imaging findings, metabolic signatures, and molecular alterations may provide information about organ damage, fibrosis, dysplasia, or future malignancy risk. These biomarkers are not proposed for broad near-term screening across all infected individuals. Their most appropriate role at present is to support research, referral decision-making, and risk stratification among selected high-risk populations where chronic pathology or persistent exposure is already evident.

These diagnostic technologies also have important limitations. Microscopy has high specificity but limited sensitivity in low-intensity infection, while antigen assays may be affected by cost, supply chains, uncertain thresholds, false positives, cross-reactivity, and interpretation challenges in very low-prevalence settings [6,7,8]. Point-of-care feasibility depends on cold-chain requirements, reagent stability, staff training, quality control, and whether results can be linked to treatment or referral decisions during the same encounter. Environmental DNA methods face additional field challenges, including heterogeneous parasite distribution in water, sampling volume, filtration logistics, DNA degradation, contamination control, assay inhibition, laboratory access, and difficulty translating a positive environmental signal into individual clinical risk [11]. These limitations reinforce the need to match diagnostic tools to the intended use: treatment decisions, elimination mapping, sentinel surveillance, or research on cancer-risk pathways.

## 3. The Infection-to-Cancer Paradigm

One of the most important advances in PNTD research is the integration of parasitology with oncology. Parasite-associated cancers should not be considered incidental outcomes of chronic infection; rather, they represent a biological continuum in which inflammation, epithelial damage, immune evasion, fibrosis, and metabolic remodeling interact over time (Table 1). This continuum is especially visible in urogenital schistosomiasis, where chronic exposure to eggs and inflammatory mediators is associated with long-term urogenital morbidity, infertility-related pathology, and malignancy-related risk [1,2,5,6].

The concept of metabolic reprogramming is relevant to this paradigm, but its role in parasite-associated human carcinogenesis should be interpreted cautiously. The Warburg effect, classically described in cancer cells as a preference for aerobic glycolysis, offers a useful hypothesis for understanding chronically inflamed parasite-associated tissue environments rather than an established clinical biomarker in PNTDs [2]. Persistent immune activation and tissue repair may create acidic, proliferative, or hypoxic microenvironments that could contribute to tumor initiation or progression, but current evidence is not sufficient to support near-term clinical risk stratification based on these metabolic features alone. Hypothesis-driven longitudinal human studies are therefore needed to determine whether specific metabolic signatures are reproducible, temporally linked to tissue progression, and predictive of clinically meaningful cancer risk after antiparasitic treatment.

Epigenetic remodeling provides a complementary but still incompletely validated mechanism. Parasite-derived molecules and chronic inflammatory signals may alter gene expression patterns in host tissues, potentially priming epithelial cells for dysplasia and malignancy [1,2,4,5]. However, most proposed epigenetic pathways remain inferential, model-based, or derived from limited human datasets. The priority should therefore be well-designed longitudinal studies in endemic populations that collect exposure history, treatment timing, tissue pathology, host genomic and epigenomic profiles, inflammatory markers, and long-term clinical outcomes. Such studies would help distinguish mechanistic plausibility from actionable biomarkers and clarify whether metabolic or epigenetic alterations should inform future surveillance, prevention, or therapeutic trials.

Cancer development in these infections is also modified by cofactors that should be measured rather than treated as background noise. Host genetics may influence inflammatory tone, epithelial repair, immune regulation, and susceptibility to fibrosis or dysplasia. Environmental exposures, including unsafe water, sanitation deficits, raw fish consumption, tobacco smoke, alcohol, aflatoxin, nitrosamines, and occupational or household contact with contaminated water, can alter both infection risk and carcinogenic potential [2,4]. Co-infections such as HIV, malaria, HBV, and HCV may further modify immune activation, liver injury, treatment response, and competing morbidity [12,13,14]. Nutrition, anaemia, microbiome composition, chronic inflammation, and delayed healthcare access influence whether parasite-associated tissue injury is detected, treated, or allowed to progress. These interacting determinants support a multimorbidity model of parasite-associated cancer risk rather than a single-pathogen deterministic model (Figure 2).

## 4. Therapeutic Innovation and Long-Term Risk Reduction

Praziquantel remains central to schistosomiasis control because it is affordable, widely used, and effective against adult schistosomes, but its limitations are important for long-term risk reduction [15,16]. It has limited activity against juvenile worms, does not prevent reinfection when environmental exposure continues, may require repeated treatment in high-transmission settings, and does not reliably reverse established fibrosis, organ damage, or premalignant tissue changes. Heavy reliance on a single drug also raises the need for pharmacovigilance and monitoring for reduced susceptibility, even though widespread clinically consequential resistance has not been established [15,16]. Treatment strategies should therefore be paired with safe water, sanitation, snail control, health education, surveillance, and risk-based clinical referral rather than framed as sufficient cancer prevention on their own.

Screening programs should be targeted and feasible. In urogenital schistosomiasis, community haematuria screening, urine microscopy or antigen testing, and ultrasound assessment of urinary tract morbidity can help identify individuals who require treatment or referral, although ultrasound protocols require training and may be difficult to implement uniformly [17]. In liver-fluke endemic regions, screening approaches may combine stool testing, exposure history, hepatobiliary ultrasound, liver-function assessment, and referral for suspected cholangiocarcinoma where regional risk is high. These programs should prioritize high-exposure communities, persistent symptoms, abnormal imaging, recurrent infection, and cofactor burden rather than attempting costly population-wide cancer screening without evidence of benefit.

Vaccine development also remains a priority, but its relationship to cancer prevention should be framed cautiously. Candidate vaccines for schistosomiasis, including those targeting key parasite antigens, are expected primarily to reduce infection, reinfection, parasite burden, and repeated exposure to egg-induced inflammation [18,19]. Any downstream reduction in fibrosis, dysplasia, or parasite-associated cancer risk would likely be indirect and should not be assumed without long-term evidence. Vaccine trials in endemic populations should therefore include extended follow-up, morbidity outcomes, inflammatory and tissue-injury biomarkers, and, where feasible, cancer-related surrogate endpoints to determine whether reduced transmission or parasite burden translates into measurable cancer-risk reduction. In parallel, the microbiome is emerging as a relevant modifier of susceptibility, immune response, and tissue injury. Understanding how gut and urogenital microbial communities interact with parasitic infections may reveal new preventive or therapeutic pathways.

## 5. Digital Health, Geospatial Intelligence, and Pathology Support

Digital innovation is transforming PNTD surveillance and diagnosis. Geospatial artificial intelligence can combine satellite imagery, climate variables, hydrology, and land-use data to predict vector or intermediate-host habitats, such as snail environments relevant to schistosomiasis transmission [20,21,22]. These tools are increasingly important as climate change alters ecological niches and expands the geography of exposure.

Artificial intelligence may also strengthen pathology workflows in endemic regions. Algorithms trained to detect parasite eggs, granulomatous lesions, dysplasia, and early malignant changes in digital biopsy slides could reduce diagnostic delays and support clinicians where specialist pathology services are scarce [23,24]. However, these tools must be validated in diverse endemic settings and integrated with ethical, explainable, and locally sustainable implementation models. Practical constraints, including data quality, internet connectivity, equipment maintenance, workforce training, local technical support, and governance of artificial intelligence tools, must be addressed before digital pathology and geospatial intelligence can be implemented equitably at scale.

## 6. Clinical Integration: From Infection History to Cancer Staging

A key priority for the coming decade is the systematic integration of parasitic exposure history into clinical evaluation. In endemic regions, a bladder tumor associated with previous urogenital schistosomiasis may differ biologically and clinically from a tumor arising in a non-endemic context [1]. Incorporating infection history, parasite burden, tissue findings, and molecular biomarkers into staging frameworks could improve prognosis and guide treatment decisions [1,2].

This approach requires closer collaboration among parasitologists, oncologists, pathologists, epidemiologists, molecular biologists, and public health practitioners. It also requires longitudinal cohorts that follow treated individuals beyond infection clearance to determine who develops fibrosis, metaplasia, or malignancy [1]. Without such follow-up, the delayed burden of PNTDs will remain underestimated.

A practical clinical pathway should be risk-stratified rather than universal. It could begin with assessment of lifetime exposure history, residence in endemic areas, recurrent infection, haematuria or hepatobiliary symptoms, and known high-risk co-exposures, followed by parasite diagnostics to determine active or previous infection. Imaging, pathology review, and molecular or metabolic biomarkers should be reserved for individuals or regions with clinical warning signs, persistent morbidity, high cumulative exposure, or established referral indications. Such a pathway would connect community-level control programs with individual-level oncology referral only when high-risk features are detected, reducing the likelihood of unnecessary resource use (Table 2).

## 7. Co-Infections and Multimorbidity in Endemic Settings

Cancer-risk pathways in endemic areas rarely occur in isolation from other infections or chronic conditions. HIV, malaria, hepatitis B virus, hepatitis C virus, bacterial causes of chronic inflammation, malnutrition, anaemia, and environmental exposures may overlap geographically with schistosomiasis and food-borne trematodiases. These conditions can modify immune activation, wound repair, microbial ecology, liver inflammation, treatment response, and access to care, thereby complicating attribution of cancer risk to a single parasite. For example, schistosomiasis has been associated with higher odds of sexually transmitted infections, while HIV–malaria co-infection can intensify immunological and clinical burden in sub-Saharan Africa [12,13].

For hepatobiliary malignancy, co-exposures are especially important because viral hepatitis, liver flukes, alcohol-related liver disease, aflatoxin exposure, and metabolic disease may converge on chronic hepatic or biliary inflammation. A systematic review of hepatitis virus and liver-fluke co-infection found limited and mixed evidence, with some studies suggesting potential interaction and others finding no clear relationship [14]. This uncertainty should be treated as a research priority rather than a reason to exclude co-morbidity from parasite-associated cancer frameworks. Future longitudinal cohorts should therefore capture HIV status, malaria exposure, HBV and HCV status, nutritional indicators, microbiome features, environmental carcinogen exposure, and treatment history so that effect modification, competing risks, and multimorbidity can be evaluated explicitly.

## 8. Implementation Science and Phased Roll-Out in Low-Resource Settings

Translation of this framework into practice will require explicit attention to implementation science, especially in low-resource endemic settings where cost, laboratory infrastructure, digital connectivity, equipment maintenance, referral capacity, and trained personnel may be limiting [28,29]. A phased roll-out should begin with strengthening existing PNTD platforms—community mapping, preventive chemotherapy, microscopy, symptom screening, sanitation, and referral pathways—before introducing higher-cost molecular diagnostics, environmental DNA surveillance, digital pathology, or geospatial artificial intelligence. This stepwise approach would allow programs to identify the minimum effective package for each setting, reserve intensive follow-up for high-risk groups, build local technical capacity, and avoid widening inequities through technologies that cannot be sustained after pilot funding ends.

Implementation should also include governance mechanisms for data ownership, privacy, algorithmic accountability, interoperability with national health information systems, and community trust. Pilot programs should be evaluated not only for diagnostic accuracy or predictive performance, but also for feasibility, acceptability, adoption, appropriateness, implementation cost, fidelity, penetration, equity, and long-term sustainability [28,29,30]. Embedding these criteria into early implementation studies would help ensure that precision diagnostics and digital surveillance complement, rather than displace, essential investments in sanitation, primary care, pathology services, and local public health capacity.

A practical prioritization framework can help distinguish near-term implementation priorities from technologies that remain primarily research-oriented. Rather than treating artificial intelligence, metabolomics, environmental DNA, vaccines, microbiome research, and molecular biomarkers as equally deployable, programs should assess each tool according to readiness for field use, infrastructure requirements, workforce burden, expected public health value, cost-effectiveness, and equity impact. Table 3 proposes a tiered recommendation to guide staged adoption in endemic and low-resource settings.

## 9. Future Directions

Future research should move from parallel advances in diagnostics, surveillance, therapeutics, and oncology toward an integrated but selective implementation agenda (Figure 3). Priority actions include validating biomarker panels that combine parasite detection, host metabolic signatures, inflammatory mediators, epigenetic alterations, early cancer-risk indicators, and co-infection profiles through hypothesis-driven longitudinal human studies; establishing longitudinal cohorts in endemic regions that are enriched for high cumulative exposure or persistent morbidity; integrating environmental surveillance with clinical registries; developing artificial intelligence pathology validation datasets that are locally representative in terms of geographic setting, population demographics, parasite species and strain diversity, specimen type, lesion stage, staining protocols, scanner or microscope platform, co-infection burden, and health-system context; and incorporating chronic pathology endpoints into therapeutic and vaccine trials [1,2,3,4,5,6,7,8,9,10,11,12,13,14,15,16,17,18,19,20,21,22,23,24,27,28,29,30]. These priorities should be sequenced according to readiness, affordability, infrastructure requirements, and expected public health value, rather than pursued as equivalent implementation targets. Such actions would support risk stratification before irreversible tissue damage occurs and could help identify the minority of individuals who remain vulnerable to chronic inflammatory, fibrotic, and malignant sequelae even after infection has been controlled [1,2,3,4,5,6,12,13,14,25,26,27,31].

This agenda also requires longitudinal cohorts in endemic regions that follow treated individuals beyond parasitological cure. These cohorts should quantify delayed outcomes such as fibrosis, metaplasia, dysplasia, organ dysfunction, and malignancy, while also linking individual-level clinical data to environmental and geospatial surveillance [10,16,17,18]. In this regard, environmental DNA monitoring, snail-host mapping, climate data, and geospatial artificial intelligence could be combined to identify persistent transmission niches and guide locally targeted interventions [10,16,17,18].

At the diagnostic and clinical interface, artificial intelligence-assisted pathology tools should be validated for parasite-associated lesions across diverse endemic settings, with careful attention to data quality, algorithm transparency, local infrastructure, and clinical accountability [23,24]. At the same time, therapeutic and vaccine trials should be redesigned so that endpoints extend beyond parasite clearance to include prevention of chronic inflammation, tissue remodeling, fibrosis, and oncogenic progression [1,2,3,4,5,6,10,15,16,17,18,19]. Finally, parasitic exposure history should be embedded into oncology and pathology workflows in endemic areas, ensuring that infection-associated cancer risk is recognized during diagnosis, staging, surveillance, and survivorship care [1,2,5,25,26,31].

## 10. Conclusions

The future of cancer-associated PNTD research and control should be proportionate, risk-stratified, and implementation-focused. Treating infection remains the highest-priority intervention for most affected individuals, and cancer prevention should be directed toward the smaller subset of people and communities with repeated exposure, persistent morbidity, chronic inflammation, co-infections, environmental cofactors, or abnormal clinical findings. Actionable priorities include strengthening preventive chemotherapy, sanitation, safe-water access, snail or reservoir control where relevant, symptom screening, and referral pathways; validating feasible diagnostics for active infection and tissue injury; embedding parasitic exposure history into pathology and oncology assessment in endemic regions; and establishing longitudinal cohorts that can distinguish mechanistic plausibility from clinically useful biomarkers. Advanced technologies such as omics platforms, environmental DNA surveillance, digital pathology, geospatial artificial intelligence, and vaccine candidates should be introduced through staged implementation studies, referral-center pilots, or research cohorts before broad deployment. Future perspectives should focus on defining minimum effective care packages for low-resource settings, identifying high-risk groups who benefit from targeted follow-up, measuring cost-effectiveness and equity impact, and integrating parasite control with long-term prevention of chronic tissue injury and parasite-associated malignancy [32].

## Figures and Tables

**Figure 1:**
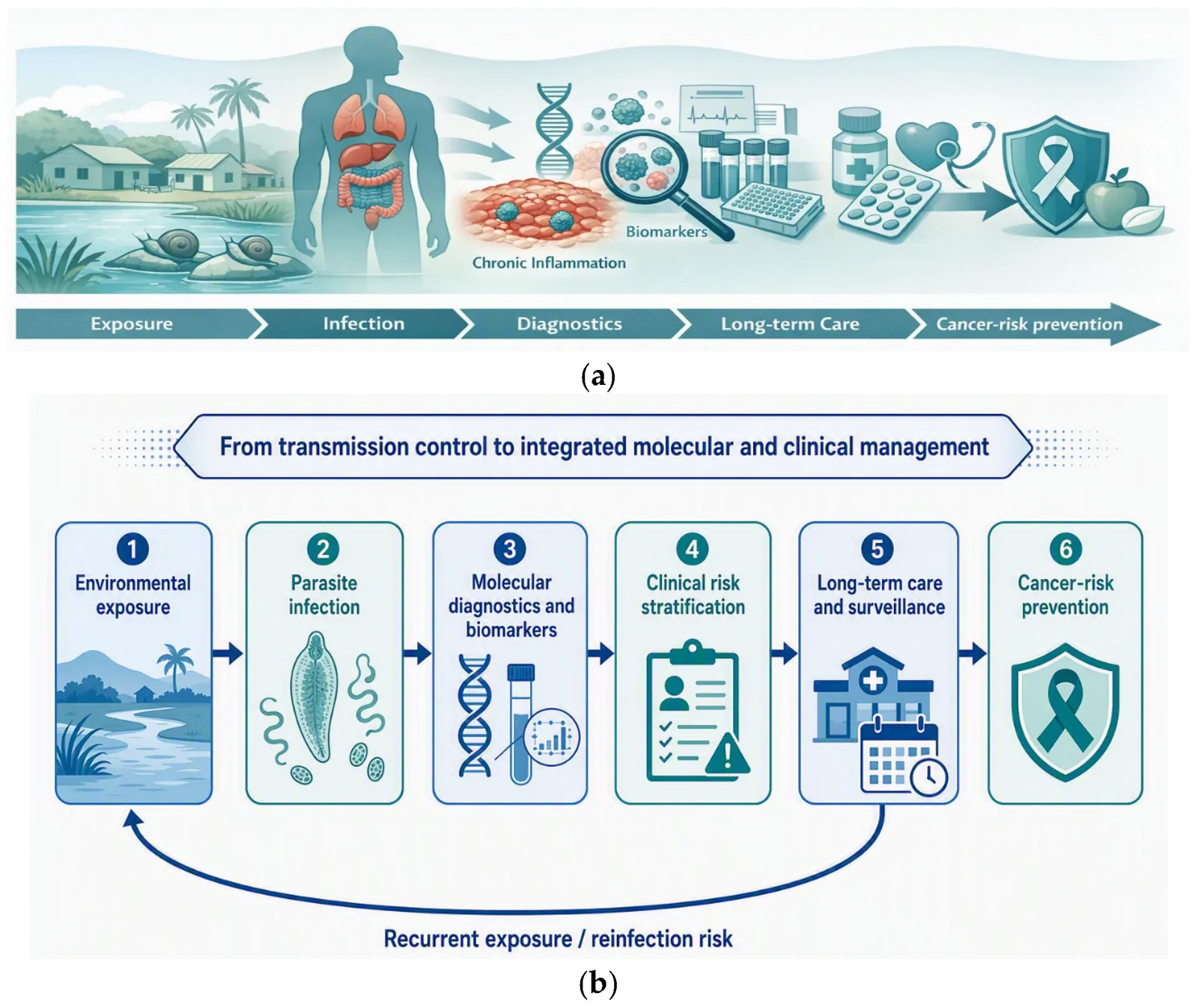
(**a**) Conceptual shift from transmission control to integrated molecular and clinical management of parasitic neglected tropical diseases. The framework links environmental exposure, infection, molecular diagnostics, long-term care, and cancer-risk prevention, Source: original figure created by the author for this Perspective. (**b**) Feedback loop from long-term care back to environmental exposure to reflect recurrent infection risk and the need for continued prevention after treatment. Source: original schematic created by the author for this Perspective.

**Figure 2:**
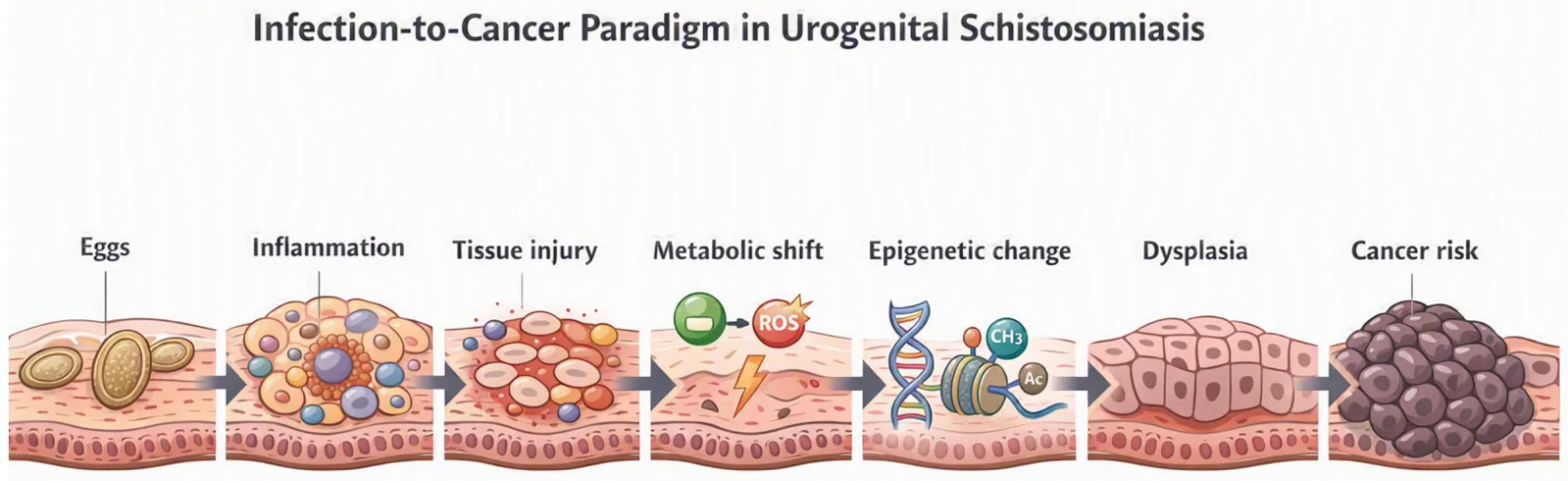
Infection-to-cancer continuum in parasite-associated malignancy. Chronic parasite-induced inflammation may promote tissue injury, metabolic reprogramming, epigenetic remodeling, dysplasia, and eventual cancer-risk progression. Source: original schematic created by the author for this Perspective.

**Figure 3:**
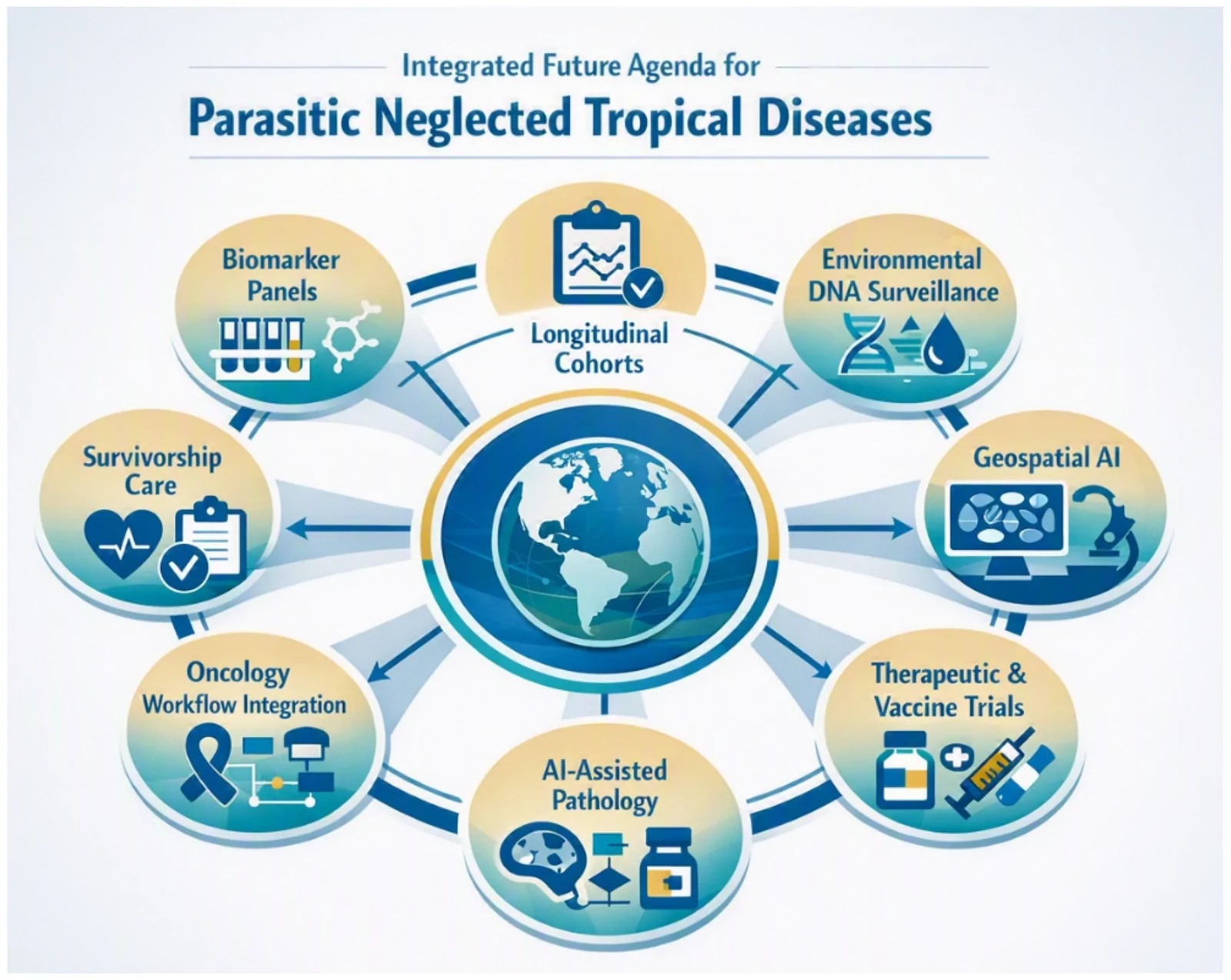
Integrated future agenda for PNTD research and care. Biomarkers, longitudinal cohorts, environmental surveillance, geospatial artificial intelligence, digital pathology, therapeutic innovation, vaccine development, and oncology integration should be connected within a unified implementation framework. Source: original schematic created by the author for this Perspective.

**Table 1:** Strength of evidence for carcinogenic mechanisms associated with selected cancer-associated parasitic neglected tropical diseases.

Parasite	Cancer Association	Mechanistic Evidence	Overall Evidence Strength
***Schistosoma haematobium***	Urinary bladder cancer, especially squamous cell carcinoma	Human epidemiology, IARC Group 1 classification, chronic egg-induced inflammation, epithelial injury, fibrosis, nitrosative/oxidative stress, and supportive experimental and molecular data	Strong for association; moderate for specific molecular pathways in humans
***Opisthorchis viverrini***	Cholangiocarcinoma, especially in endemic regions of Southeast Asia	Human epidemiology, IARC Group 1 classification, chronic biliary inflammation, epithelial hyperplasia, periductal fibrosis, parasite excretory-secretory products, oxidative stress, and interaction with dietary or environmental cofactors	Strong for association; moderate-to-strong for inflammation-mediated mechanisms
***Clonorchis sinensis***	Intrahepatic and extrahepatic cholangiocarcinoma	Human epidemiology, IARC Group 1 classification, chronic ductal irritation, inflammation, epithelial proliferation, fibrosis, molecular alterations, and biomarker studies, with fewer longitudinal human datasets than for some endemic *O. viverrini* settings	Strong for association; moderate for detailed causal pathways and validated biomarkers

**Table 2:** Parasite-associated chronic pathology, cancer links, diagnostic tools, and clinical or research priorities for selected parasitic neglected tropical diseases.

Parasite and Disease Context	Affected Tissue and Chronic Pathology	Cancer Association	Diagnostic or Surveillance Tools	Clinical or Research Priority
*Schistosoma haematobium* Urogenital schistosomiasis	Bladder and urogenital tract Egg-induced inflammation, fibrosis, epithelial injury, and dysplasia	Bladder cancer, particularly squamous cell carcinoma of the urinary bladder; IARC Group 1 carcinogen. Reported relative risks for urinary bladder cancer range from approximately 2 to 14 across epidemiological studies [1,25,26].	Urine microscopy; circulating antigens; imaging; pathology; metabolic biomarkers; eDNA surveillance	Link parasite control with targeted bladder evaluation only for high-risk individuals or settings, rather than universal long-term surveillance
*Opisthorchis viverrini* Food-borne trematodiasis	Bile ducts and hepatobiliary system Chronic biliary inflammation, epithelial injury, and fibrosis	Biliary tract cancer, especially intrahepatic/perihilar cholangiocarcinoma in endemic regions. A recent meta-analysis reported an odds ratio of 3.69 for cholangiocarcinoma associated with *O. viverrini* infection [27].	Stool diagnostics; imaging; molecular biomarkers; geospatial risk mapping	Integrate infection detection with hepatobiliary cancer prevention and early diagnosis only where exposure intensity, symptoms, imaging, or regional risk justify referral
*Clonorchis sinensis* Food-borne trematodiasis	Bile ducts and hepatobiliary system Chronic epithelial irritation, inflammation, and ductal remodeling	Biliary tract cancer, especially cholangiocarcinoma of the intrahepatic and extrahepatic bile ducts. A recent meta-analysis reported an odds ratio of 4.49 for cholangiocarcinoma associated with *C. sinensis* infection [27].	Stool diagnostics; imaging; serology or molecular tools; environmental surveillance	Define selective follow-up pathways for high-risk endemic populations and avoid population-wide intensive surveillance without evidence of benefit

**Table 3:** Tiered prioritization matrix for emerging technologies in PNTD control, diagnostics, surveillance, and cancer-risk prevention.

Technology or Intervention	Readiness Level	Cost and Infrastructure Profile	Tiered Recommendation
**Preventive chemotherapy, microscopy, symptom screening, exposure history, and referral pathways**	High readiness; already embedded in many control programs	Lowest relative cost; uses existing primary care, community health, and laboratory platforms	Tier 1: universal foundation for endemic settings; strengthen before adding complex platforms
**Circulating antigen tests, targeted imaging, pathology review, and structured cancer-risk referral**	Moderate-to-high readiness depending on setting and disease target	Moderate cost; requires supply chains, trained personnel, referral capacity, and quality assurance	Tier 2: priority for regional referral hubs and high-burden districts with known chronic morbidity or cancer risk
**Environmental DNA surveillance and geospatial risk mapping**	Moderate readiness; promising for hotspot detection but implementation varies by ecology and laboratory capacity	Moderate-to-high cost; requires sampling systems, molecular laboratories, geospatial expertise, and data integration	Tier 2–3: deploy first as sentinel surveillance in elimination or recrudescence-prone areas before national scale-up
**Artificial intelligence for digital pathology and geospatial prediction**	Developing readiness; strong potential but dependent on local validation and governance	High infrastructure burden; requires scanners or imaging devices, connectivity, maintenance, representative datasets, and accountability frameworks	Tier 3: evaluate through implementation studies and referral-center pilots before broad deployment
**Metabolomics, epigenomics, microbiome profiling, and multi-omics cancer-risk biomarkers**	Early-to-moderate readiness; valuable for discovery and risk stratification but not yet routine public health tools	High cost and technical complexity; requires advanced laboratories, biobanking, longitudinal cohorts, and bioinformatics	Tier 3: prioritize for research cohorts, biomarker validation, and high-risk clinical pathways rather than population-wide roll-out
**Vaccines and host-directed or adjunctive therapeutics**	Variable readiness; several candidates remain in preclinical or early clinical development	Potentially high development and delivery costs; cost-effectiveness depends on efficacy, durability, the target population, and integration with chemotherapy	Tier 3: maintain as strategic research priorities while evaluating long-term endpoints beyond parasite clearance

## Data Availability

No new data were created or analyzed in this study. Data sharing is not applicable to this article.

## References

[1] Rather S.A., Wani Z.A., Mustafa R.A., Bharti P., Kousar R., Ashraf M.V., Ahmad S., Shah A.A., Khan M.A.H. (2025). Carcinogenic parasites: Insights into the epidemiology and possible mechanisms of cancer. Mutagenesis.

[2] Espinoza J.L. (2023). Fluke-related cholangiocarcinoma: Challenges and opportunities. Pathogens.

[3] World Health Organization (2026). Schistosomiasis. Fact Sheet.

[4] Qian M.-B., Keiser J., Utzinger J., Zhou X.-N. (2024). Clonorchiasis and opisthorchiasis: Epidemiology, transmission, clinical features, morbidity, diagnosis, treatment, and control. Clin. Microbiol. Rev..

[5] Huang C., Wu Y., Wu A., Huang L., Fang S., Xu C., Liu T., Li Y., Li X. (2026). *Clonorchis sinensis* and cholangiocarcinoma: Molecular mechanisms and biomarker advances. Parasite.

[6] Marques R.P., Ahmad W., Soares R., Oliveira K.C., Botelho M.C. (2024). Insights into the state of the art of urogenital schistosomiasis with a focus on infertility. Trop. Med. Infect. Dis..

[7] Vaillant M.T., Philippy F., Neven A., Barré J., Bulaev D., Olliaro P.L., Utzinger J., Keiser J., Garba A.T. (2024). Diagnostic tests for human *Schistosoma mansoni* and *Schistosoma haematobium* infection: A systematic review and meta-analysis. Lancet Microbe.

[8] Hoekstra P.T., de Dood C.J., Abdoel T., Hilt S., van Diepen A., Polman K., Kremsner P., van Lieshout L., Kreidenweiss A., Adegnika A.A. (2024). Detecting two *Schistosoma* circulating antigens—CCA and CAA—In urine and serum to improve diagnosis of human schistosomiasis. Front. Parasitol..

[9] Ally O., Kanoi B.N., Ochola L., Nyanjom S.G., Shiluli C., Misinzo G., Gitaka J. (2024). Schistosomiasis diagnosis: Challenges and opportunities for elimination. PLoS Negl. Trop. Dis..

[10] Chienwichai P., Tipthara P., Tarning J., Limpanont Y., Chusongsang P., Chusongsang Y., Kiangkoo N., Adisakwattana P., Reamtong O. (2024). Identification of trans-genus biomarkers for early diagnosis of intestinal schistosomiasis and progression of gut pathology in a mouse model using metabolomics. PLoS Negl. Trop. Dis..

[11] Revolteado M.J., Sato M.O., Valencia J., Jiz M., Cervantes E., Aniceto R., Inobaya M., Gray D., Gordon C.A., Cai P. (2025). Seasonality and dynamics of schistosomiasis in the environment: Usefulness of environmental DNA (eDNA) surveillance system at a community level for risk mapping schistosomiasis in Ekiran Village, Philippines. mSphere.

[12] Asare K.K., Afful P., Abotsi G.K., Adu-Gyamfi C.O., Benyem G., Katawa G., Arndts K., Ritter M. (2024). Schistosomiasis endemicity and its role in sexually transmitted infections: A systematic review and meta-analysis. Front. Parasitol..

[13] Figueroa-Romero A., Saura-Lázaro A., Fernández-Luis S., González R. (2024). Uncovering HIV and malaria interactions: The latest evidence and knowledge gaps. Lancet HIV.

[14] O’Rourke A. (2024). A systematic review of the effects of hepatitis B and C virus on the progression of liver fluke infection to liver cancer. Trop. Dis. Travel Med. Vaccines.

[15] Eastham G., Fausnacht D., Becker M.H., Gillen A., Moore W. (2024). Praziquantel resistance in schistosomes: A brief report. Front. Parasitol..

[16] Alwan S.N., Taylor A.B., McHardy S.F., Cameron M.D., LoVerde P.T. (2025). Development of a novel compound effective against juvenile, adult, and drug-resistant *Schistosoma* species. Pharmaceutics.

[17] Manciulli T., Marangoni D., Salas-Coronas J., Bocanegra C., Richter J., Gobbi F., Motta L., Minervini A., Bartoloni A., Zammarchi L. (2023). Diagnosis and management of complicated urogenital schistosomiasis: A systematic review of the literature. Infection.

[18] Kim J., Davis J., Lee J., Cho S.-N., Yang K., Yang J., Bae S., Son J., Kim B., Whittington D. (2024). An assessment of a GMP schistosomiasis vaccine (SchistoShield^®^). Front. Trop. Dis..

[19] Houlder E.L., Ferreira da Silva L., van Diepen A., Sena Amaral M., Wilson R.A., Hokke C.H., Roestenberg M., Bakker W.A.M. (2025). Pre-clinical studies of *Schistosoma mansoni* vaccines: A scoping review. PLoS Negl. Trop. Dis..

[20] Grover E.N., Allshouse W.B., Lund A.J., Liu Y., Paull S.H., James K.A., Crooks J.L., Carlton E.J. (2023). Open-source environmental data as an alternative to snail surveys to assess schistosomiasis risk in areas approaching elimination. Int. J. Health Geogr..

[21] Asare K.K., Mohammed M.-D.W., Aboagye Y.O., Arndts K., Ritter M. (2025). Impact of climate change on schistosomiasis transmission and distribution—Scoping review. Int. J. Environ. Res. Public Health.

[22] Cunningham L.J., Nkolokosa C., Risse M., Makaula P., Archer J., Namacha G., Chammudzi P., Kapira D., Lally D., Ntaba B.P. (2026). Tracking the spatial and longitudinal dynamics of mixed infections of urogenital and intestinal schistosomiasis, inclusive of *Schistosoma mattheei*, in two sentinel rural communities from southern Malawi. Philos. Trans. R. Soc. B Biol. Sci..

[23] Ward P.K., Mohammed M.A., Ayana M., Broadfield L.A., Dahlberg P., Dana D., Leta G., Mekonnen Z., Nabatte B., Kabatereine N. (2026). An artificial intelligence-powered digital pathology platform to support large-scale deworming programs against soil-transmitted helminthiasis and intestinal schistosomiasis in resource-limited settings. PLoS Negl. Trop. Dis..

[24] Rubio Maturana C., Dantas de Oliveira A., Zarzuela F., Ruiz E., Sulleiro E., Mediavilla A., Martínez-Vallejo P., Nadal S., Pumarola T., López-Codina D. (2024). Development of an automated artificial intelligence-based system for urogenital schistosomiasis diagnosis using digital image analysis techniques and a robotized microscope. PLoS Negl. Trop. Dis..

[25] Lingscheid T., Kurth F., Clerinx J., Marocco S., Trevino B., Gobbi F., Bottieau E., Hatz C., Zammarchi L., Richter J. (2023). Schistosomiasis-associated bladder pathology and cancer risk: Current diagnostic and clinical perspectives. Travel Med. Infect. Dis..

[26] International Agency for Research on Cancer (2024). Infection with carcinogenic parasites and cancer prevention priorities: Recent evidence update. IARC Evidence Summary.

[27] Huang Y.-L., Zhang K.-Y., Sun Y.-L., Qian M.-B., Wang Z. (2024). The risk of hepatobiliary complications in Clonorchis and Opisthorchis infection: A systematic review and meta-analysis. Acta Trop..

[28] The Lancet Global Health (2023). Implementing implementation science in global health. Lancet Glob. Health.

[29] Proctor E.K., Bunger A.C., Lengnick-Hall R., Gerke D.R., Martin J.K., Phillips R.J., Swanson J.C. (2023). Ten years of implementation outcomes research: A scoping review. Implement. Sci..

[30] Pérez Jolles M., Fort M.P., Glasgow R.E. (2024). Aligning the planning, development, and implementation of complex interventions to local contexts with an equity focus: Application of the PRISM/RE-AIM framework. Int. J. Equity Health.

[31] Sithithaworn P., Andrews R.H., Petney T.N., Saijuntha W., Laummaunwai P., Sripa B. (2024). Liver fluke infection and cholangiocarcinoma in endemic regions: Updated epidemiology and prevention priorities. Curr. Trop. Med. Rep..

[32] Khurana S., Singh S., Malla N. (2025). Parasitic infections and cancer: Emerging mechanisms, diagnostic gaps, and future research directions. Infect. Genet. Evol..

